# Kisspeptin/Neurokinin B/Dynorphin (KNDy) cells as integrators of diverse internal and external cues: evidence from viral-based monosynaptic tract-tracing in mice

**DOI:** 10.1038/s41598-019-51201-0

**Published:** 2019-10-14

**Authors:** Aleisha M. Moore, Lique M. Coolen, Michael N. Lehman

**Affiliations:** Brain Health Research Institute and Dept. of Biological Sciences, Kent State University, Kent, OH USA

**Keywords:** Neural circuits, Neuroendocrine diseases

## Abstract

Neurons in the hypothalamic arcuate nucleus (ARC) that co-express kisspeptin, neurokinin B and dynorphin (KNDy cells) are essential for mammalian reproduction as key regulators of gonadotropin-releasing hormone (GnRH) secretion. Although multiple endogenous and exogenous signals act indirectly via KNDy neurons to regulate GnRH, the identity of upstream neurons that provide synaptic input to this subpopulation is unclear. We used rabies-mediated tract-tracing in transgenic *Kiss1-*Cre mice combined with whole-brain optical clearing and multiple-label immunofluorescence to create a comprehensive and quantitative brain-wide map of neurons providing monosynaptic input to KNDy cells, as well as identify the estrogen receptor content and peptidergic phenotype of afferents. Over 90% of monosynaptic input to KNDy neurons originated from hypothalamic nuclei in both male and female mice. The greatest input arose from non-KNDy ARC neurons, including proopiomelanocortin-expressing cells. Significant female-dominant sex differences in afferent input were detected from estrogen-sensitive hypothalamic nuclei critical for reproductive endocrine function and sexual behavior in mice, indicating KNDy cells may provide a unique site for the coordination of sex-specific behavior and gonadotropin release. These data provide key insight into the structural framework underlying the ability of KNDy neurons to integrate endogenous and environmental signals important for the regulation of reproductive function.

## Introduction

Reproductive capacity is dependent on the pulsatile secretion of the gonadotrophin luteinizing hormone (LH), which is driven by the pulsatile release of gonadotrophin-releasing hormone (GnRH) from GnRH neurons in the hypothalamus^1^. The alteration of pulsatile LH release by a variety of internal and external stimuli acting at the hypothalamus can lead to infertility. For example, changes in insulin and leptin action within the hypothalamus during periods of nutritional insufficiency suppresses pulsatile LH release^2–5^. Conversely, in women with polycystic ovarian syndrome, the inability of gonadal steroid hormones to suppress increased pulsatile LH secretion suggests a state of impaired negative feedback^6–8^. As GnRH neurons themselves do not express the receptors required to relay metabolic and steroid hormone signals, upstream populations are likely involved.

One upstream population of cells in the arcuate nucleus (ARC) that co-express the neuropeptides kisspeptin, neurokinin B and dynorphin, known as KNDy cells, are proposed to form the long elusive GnRH/LH pulse generator^9^. As such, they provide an ideal nodal point through which peripheral signals can alter gonadotropin secretion and reproductive capacity. Consistent with this hypothesis, KNDy neurons have been implicated in a number of reproductive and non-reproductive process. This includes steroid hormone feedback control of gonadotropin secretion^10^, the effects of metabolic^11,12^ and stress-induced cues^13,14^ on reproduction, seasonal breeding^15–18^, puberty^19–21^ and the influence of gonadal steroids on prolactin^22^ and thermoregulation^23–25^. However, the mechanisms through which KNDy neurons regulate these processes are unclear.

The expression of kisspeptin and neurokinin B within KNDy neurons is modulated by gonadal steroid levels^10,26–28^ and nutritional status^29–31^. These cues were predicted to act directly at KNDy cells as they express receptors for the gonadal steroid hormones estrogen, progesterone and testosterone^10,32–35^ in addition to the metabolic hormones insulin^36^ and leptin^37^. However, although the knockout of estrogen receptor alpha (ERα) from kisspeptin neurons is sufficient to impair negative feedback in pre-pubertal animals^38^, post-pubertal deletion does not impair negative feedback^38,39^ although a moderate increase in LH pulse frequency has been measured^40^. This may signify the recruitment of neurons into the KNDy network for complete transmission of estrogen negative feedback information. Similarly, kisspeptin-specific deletion of the insulin^36^ or leptin^37^ receptor does not affect puberty onset and reproductive capacity in mice, indicating transmission through upstream populations. Consistent with this hypothesis, KNDy neurons express receptors for neurochemicals that regulate gonadotropin release using indirect circuits. This includes corticotropin-releasing hormone (CRH), which mediates the effects of stress-induced molecules on gonadotropin secretion^41^ and, neuropeptides that mediate metabolic cues in the brain, including proopiomelanocortin (POMC)/cocaine- and amphetamine-regulated transcript (CART)^42^, agouti-related peptide (AGRP)/neuropeptide Y (NPY)^43^ and pituitary adenylate cyclase-activating peptide (PACAP)^23,28^.

Therefore, afferent neuronal populations are likely critical for the physiological functioning of KNDy cells as a key node to integrate a variety of internal and external cues regulating gonadotropin release. Despite studies beginning to explore the anatomical location of afferents to KNDy cells in a small number of populations containing previously identified neurochemicals of interest^44,45^ there is limited data providing a complete brain-wide set of monosynaptic inputs to KNDy neurons. Further, the possibility of sexual dimorphism in primary afferents is unexplored despite sex-specific gonadotropin release and reproductive functions^46^. Therefore, a comprehensive map of afferents populations to KNDy neurons and identification of their neurochemical phenotype in both males and females is essential in determining the role of each population in reproductive and non-reproductive functions. To address these questions we used a powerful viral-tract tracing approach using the engineered rabies glycoprotein-deleted virus (RVDG)^47^ with male and female Kiss1-Cre mice. When combined with Cre-dependent adeno-associated viruses containing the TVA receptor to infer infection of RVDG and the rabies glycoprotein for transsynaptic spread, this system has the unique ability to provide a complete brain-wide map of cell populations with primary synaptic input specifically to KNDy cells. To explicitly define all brain regions containing virally-identified afferents, including populations with small numbers of cells, we combined rabies-mediated tract-tracing with optical tissue clearing and light sheet microscopy in the intact mouse brain^48^. Afferent populations were further quantified in sectioned tissue to identify sex differences at subnuclei resolution. Finally, using a shorter transfection period to avoid toxicity induced by the rabies virus, we determined peptidergic phenotypes of afferent populations to KNDy cells and identified subpopulations that respond to steroid hormone signals.

## Results

### Verification of KNDy neuron viral transfection

**Figure 1:**
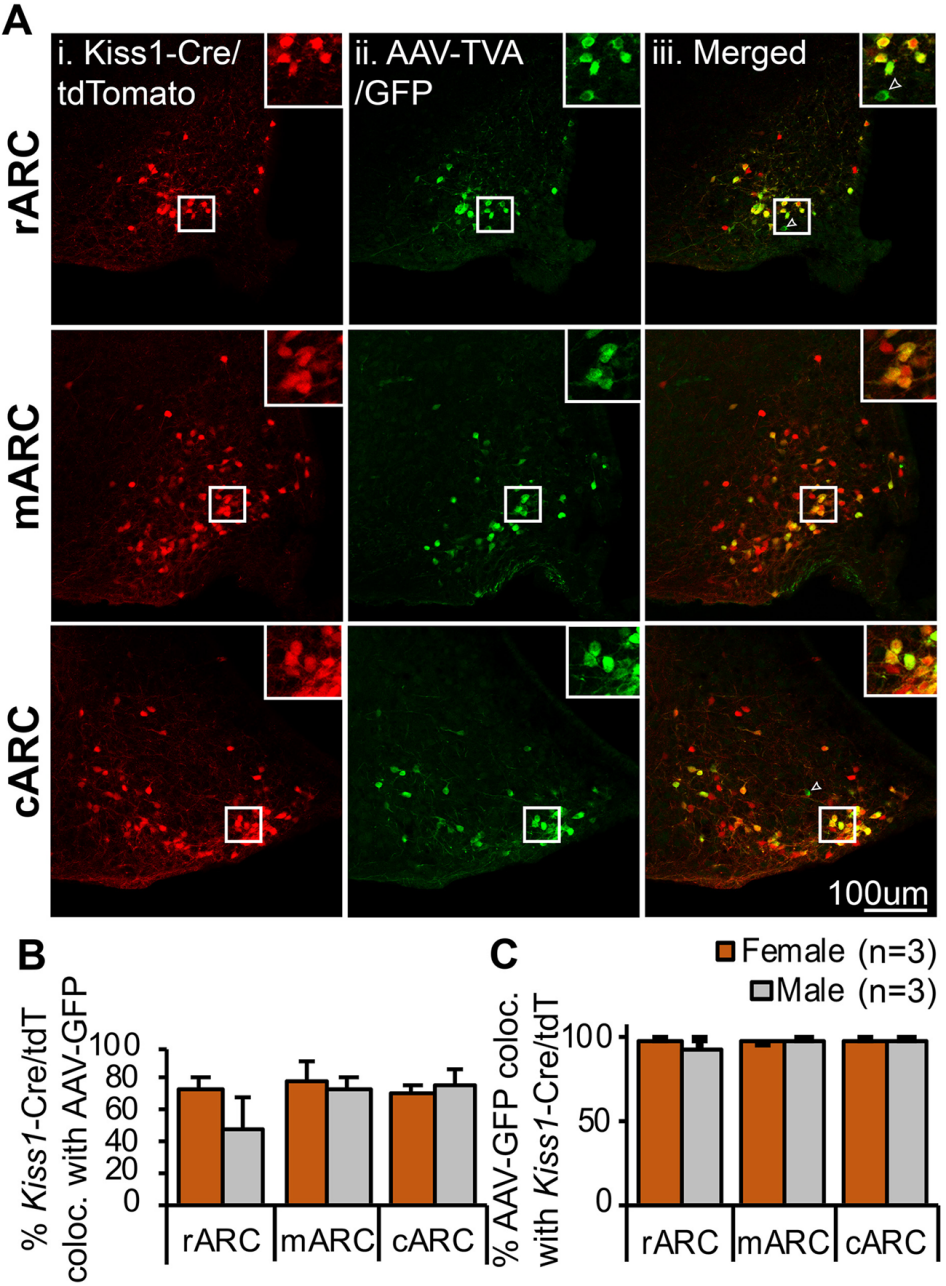
AAV viral vectors are highly specific for Cre-expressing kisspeptin neurons. (**A**) Representative confocal images of the rostral (rARC), middle (mARC) and caudal (cARC) arcuate nucleus containing Kiss1-Cre cells expressing tdTomato (i), cells immunoreactive for GFP (AAV-TVA/GFP) and detected using a secondary antibody at 650 nm pseudocolored in green (ii) and merged images (iii) with insets illustrating the high degree of colocalization between channels. Arrow points to a rare non-kisspeptin/tdTomato cell transfected with AAV-TVA/GFP. (**B**) Histogram depicting the percentage of r-cARC kisspeptin neurons transfected by AAV-TVA/GFP, as shown by the mean ± SEM percentage of Kiss1-Cre/tdTomato positive cells colocalized with AAV-GFP. (**C**) Histogram depicting the high specificity of AAV-TVA/GFP for Cre, as shown by the mean ± SEM percentage of GFP-immunoreactive cells (AAV-GFP) colocalized with Kiss1-Cre/tdTomato-positive neurons in the r-cARC. No significant differences in the specificity of viruses or the percentage of tdTomato cells transfected were detected between male and female mice in the r-cARC (Student’s t-test).

To permit transfection of KNDy neurons and synaptically-connected primary afferents, the Cre-dependent AAV viruses 2AAV8-EF1a-FLEX-GT (AAV-TVA/GFP) and 2AAV8-CAG-FLEX-oG-WPRE-SV40-PA (AAV-oG, optimized rabies glycoprotein) were stereotaxically injected into the ARC of Kiss1-Cre mice followed 7 days later by the EnVA-pseudotyped RVDG containing the fluorescent reporter mCherry. To characterize transfection of the ARC Kiss1-Cre (putative KNDy) arcuate population and confirm the specificity of Cre-dependent AAVs, colocalization of AAV-TVA/GFP with ARC Kiss1-Cre cells was used. As the KNDy peptides are difficult to visualize in the ARC of mice using immunolabelling, the percentage of Cre-positive kisspeptin cells transfected by AAV-TVA/GFP was first determined through injection of AAV vectors into the ARC of Kiss1-Cre^+/−^/tdTomato^+/−^ reporter mice (n = 3 male, n = 3 female). AAV-TVA/GFP was detected using a secondary fluorescent antibody in the 650 nm wavelength to prevent false positive co-expression of tdTomato with GFP caused by potential overlap in emission wavelengths (Fig. 1A). Of the tdTomato-positive Kiss1-Cre cells in the rostral, middle and caudal regions of the ARC, between 48–79% were transfected by the AAV-TVA/GFP virus (Fig. 1B). Higher co-expression (between 72–79%) was detected in the middle and caudal regions of the ARC compared to the rostral region, indicating the transfection of ARC Kiss1-Cre cells by AAV-TVA/GFP correlated with injection sites in the middle and caudal ARC (Supplementary Fig. 1). Of virally-transfected AAV-TVA/GFP neurons in the rostral, middle and caudal ARC, over 97% were colocalized with tdTomato in female mice, and over 92% in males (Fig. 1C), supporting transfection by AAV’s were highly specific to Cre-positive cells. No significant differences in the percentage of tdTomato-positive Kiss1-Cre cells colocalized with AAV-TVA/GFP (Fig. 1B) or the percentage of AAV-TVA/GFP-positive cells colocalized with tdTomato-positive Kiss1-Cre cells (Fig. 1C) were detected between male and female mice.

It is difficult to immunolabel for the rabies glycoprotein in order to characterize transfection of ARC Kiss1-Cre cells by AAV-oG. However, mCherry was not detected in the brain of Kiss1-Cre^+/−^ mice transfected with either AAV-TVA/GFP only (n = 4) or AAV-oG only (n = 4), supporting that both viruses are required for RVDG-mCherry expression. Finally, GFP and mCherry were not detected in wildtype Kiss1-Cre^−/−^ littermates (0 ± 0 mCherry cells, n = 5), confirming that RVDG transfection did not occur in the absence of Cre in Kiss1 cells.

### Distribution of primary afferents to KNDy neurons in the mouse brain

**Figure 2:**
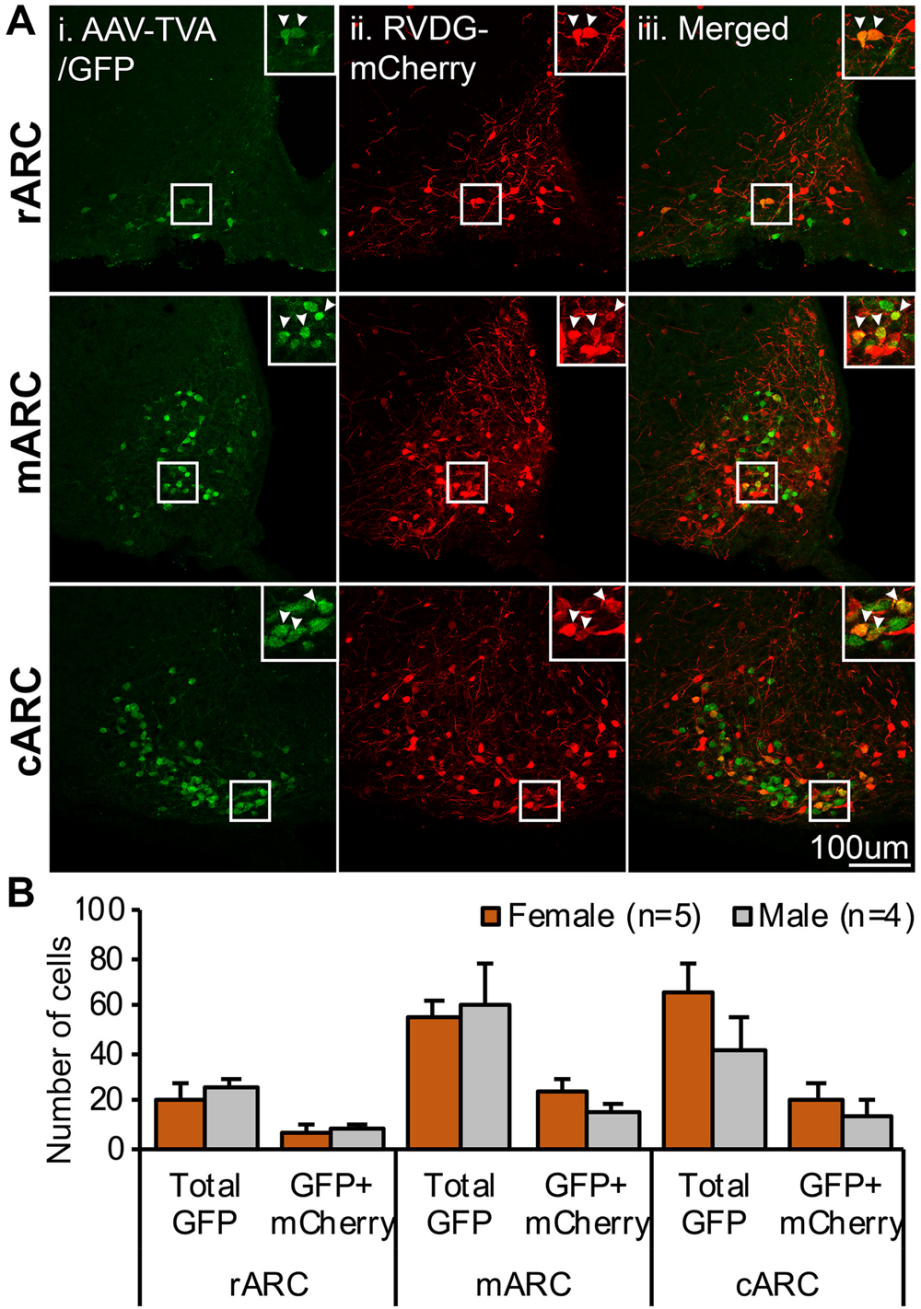
Transfection of ARC Kiss1-Cre (KNDy) cells with RVDG viral vector for monosynaptic tracing. (**A**) Representative confocal images of Kiss1-Cre cells in the rostral (rARC), middle (mARC) and caudal (cARC) arcuate nucleus transfected with AAV-TVA/GFP (i), RVDG-mCherry (ii) and merged images (iii). Insets illustrating examples of cells that co-express AAV-TVA/GFP and RVDG-mCherry (arrows). (**B**) Histogram depicting the number of cells in the rARC, mARC and cARC transfected with AAV-TVA/GFP (total GFP) and the number of cells transfected with both AAV-TVA/GFP and RVDG-mCherry (GFP + mCherry). No significant differences in the number of GFP or GFP + mCherry transfected cells were detected between male and female mice in the rostral to caudal extent of the ARC (two-way ANOVA).

For tract-tracing of afferent populations to KNDy cells, AAV and RVDG viral vectors were injected into the ARC of Kiss1-Cre^+/−^ mice and transfection quantified in sectioned tissue. As in the verification study, AAV-TVA/GFP-positive Kiss1-Cre cells colocalized with mCherry-positive RVDG transfected cells were detected within the ARC (Fig. 2A,B). These cells either represent KNDy starter cells, or, due to possible reciprocal connections between KNDy neurons, presynaptic KNDy cells. GFP and mCherry co-labeled cells were detected both on the ipsilateral (Fig. 2A) and contralateral (Supplementary Fig. 2) side of injection. The total number of Kiss1-Cre cells transfected with GFP and the total number of GFP-positive cells colocalized with mCherry was not significantly different in the rostral to caudal extent of the ipsilateral ARC between males and female mice (Fig. 2B).

**Figure 3:**
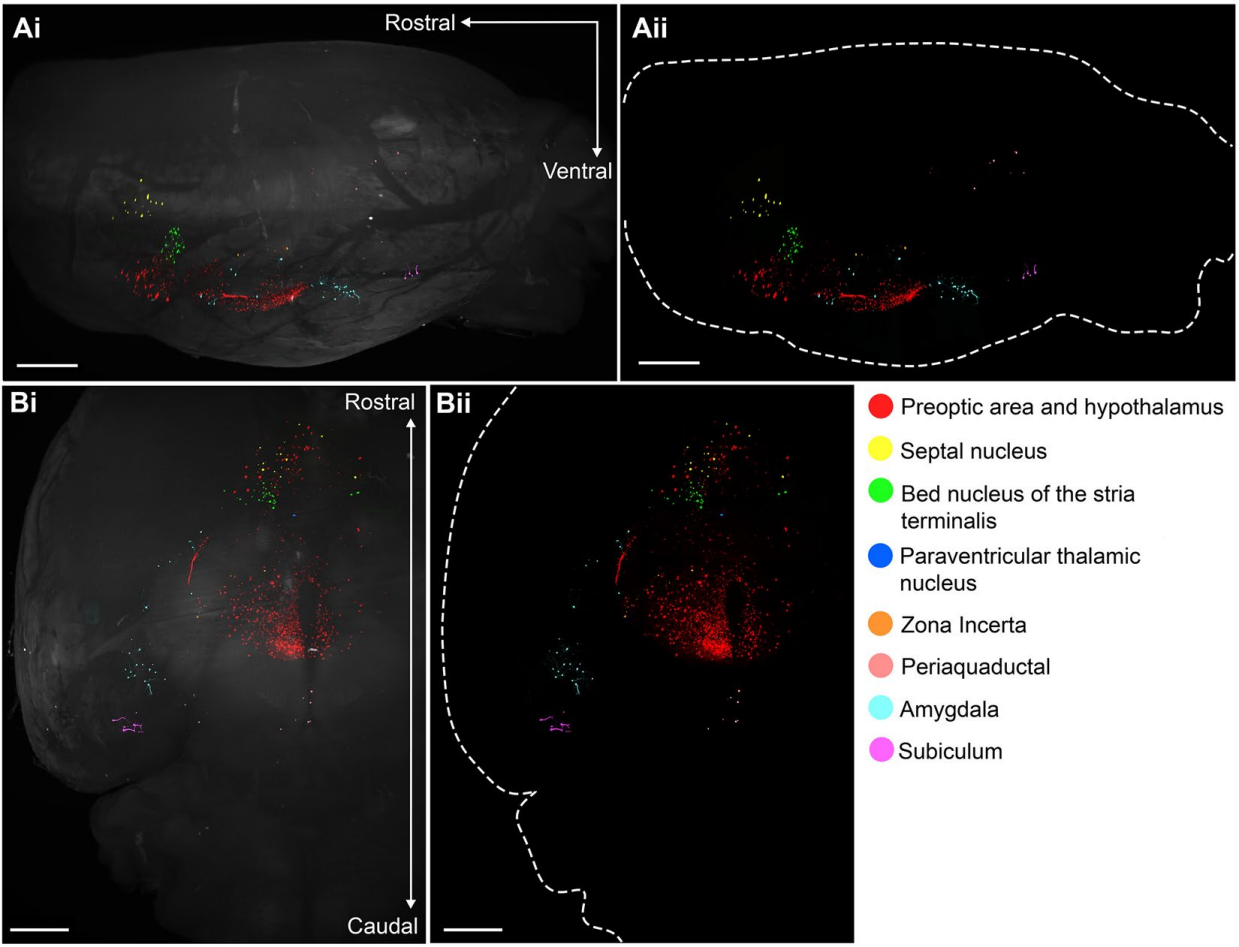
Brain-wide distribution of presynaptic KNDy neurons as visualized with 3D imaging of the intact mouse brain. The distribution of mCherry-labelled presynaptic KNDy neurons in the intact mouse brain following optical tissue clearing and light sheet microscope imaging. 3D rendering of mCherry-immunoreactivity in the mouse brain using IMARIS software, as viewed from the sagittal plane (**A**) and ventral surface (**B**). Rendering of mouse brain with (i) and without (ii) autofluorescence (488 nm laser excitation). Scale bars = 300 µm.

mCherry-labelled presynaptic cells to KNDy neurons were mapped throughout the brain. A qualitative brain-wide assessment of mCherry distribution was undertaken using immunofluorescent labelling in intact brains rendered transparent using solvent-based tissue clearing and imaging by light-sheet microscopy (Video 1, Fig. 3). The majority of mCherry-positive afferents to KNDy cells were observed within the preoptic area and hypothalamus, however, cells were also present within the bed nucleus of the stria terminalis (BNST), septal nucleus and medial amygdala. Smaller mCherry-transfected populations consisting of a few cells were detected within the zona incerta of the subthalamus, paraventricular thalamic nucleus, periaquaductal grey and the subiculum of the hippocampal formation (Video 1, Fig. 3).

**Figure 4:**
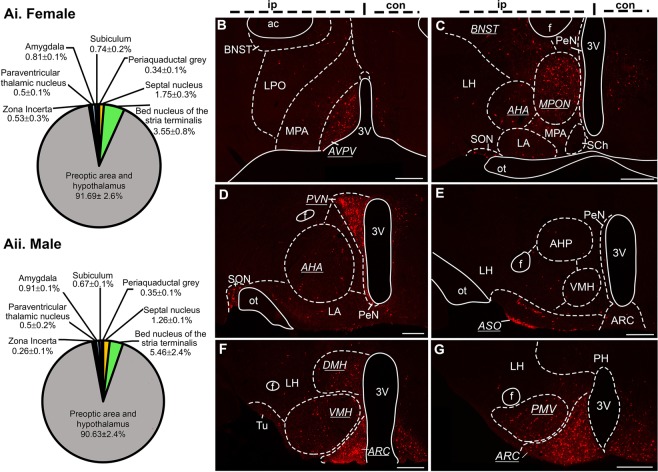
The majority of presynaptic KNDy neurons are located within hypothalamic nuclei. (**A**) The distribution of mCherry-labelled presynaptic KNDy neurons in the brain of female (i) and male (ii) mice as expressed as the mean ± SEM percentage of total mCherry-labelled cells per region per animal. (**B**–**G**) Epifluorescent images from a representative male mouse of mCherry-labelled cells in coronal brain sections. Brain nuclei that contain over 5% of total presynaptic input to KNDy neurons are underlined and italicized. Scale bars = 250 µm. AVPV = anteroventral periventricular nucleus, BNST = bed nucleus of the stria terminalis, LPO = lateral preoptic area, MPA = medial preoptic area, MPON = medial preoptic nucleus, AHA = anterior hypothalamic area, LH = lateral hypothalamus, LA = lateroanterior hypothalamus, SCh = suprachiasmatic nucleus, PVN = paraventricular nucleus, PeN = periventricular nucleus, SON = supraoptic nucleus, ASO = accessory groups of the supraoptic nucleus, VMH = ventromedial hypothalamus, DMH = dorsomedial hypothalamus, ARC = arcuate nucleus, Tu = tuberal nucleus, PH = posterior hypothalamus, PMV = ventral premammillary nucleus, f = fornix, 3 V = third ventricle.

mCherry-labeled presynaptic KNDy neurons were quantified throughout the brain on the ipsilateral side of injection in sectioned tissue from female and male mice, and the number of mCherry-positive cells in each nuclei (Supplementary Table 1) expressed as a percentage of the total mCherry-positive cells in the brain of each animal (Fig. 4A, Supplementary Table 2) in order to normalize for differences in RVDG transfection between animals. No significant difference in total ipsilateral mCherry-positive cells were detected between female (587.3 ± 109.9) and male (737.13 ± 83.8) mice (p = 0.33). The largest source of presynaptic input to KNDy neurons in both male and female mice originated from local non-GFP expressing neurons within the ARC (Fig. 4F,G). Due to the absence of GFP, these cells likely represent non-KNDy ARC neurons. Following ARC populations, regions displaying the highest density of afferent input to KNDy cells were the paraventricular nucleus (PVN, Fig. 4D), the dorsomedial hypothalamus (DMH, Fig. 4F), the anteroventral periventricular nucleus (AVPV, Fig. 4B), and the medial preoptic nucleus (MPON, Fig. 4C). A complete list of afferents within hypothalamic nuclei can be found in Supplementary Tables 1 and 2.

Although mCherry-labelled cells were observed within the periaqueductal grey of the midbrain of all female and male mice (Fig. 2, Supplementary Table 1), mCherry-positive cells in other midbrain and brainstem nuclei were only observed in a percentage of male and female mice assessed (Supplementary Fig. 3).

### Sexually dimorphic input to KNDy neurons

**Figure 5:**
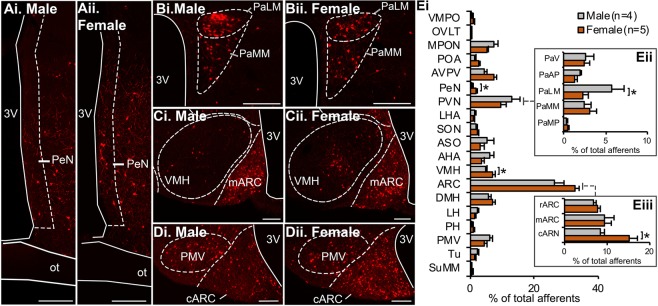
Sexually differentiated synaptic input to KNDy neurons from hypothalamic nuclei. Representative epifluorescent images of sections from male (i) and female (ii) mice containing the periventricular nucleus (PeN, **A**), lateral magnocellular part of the paraventricular nucleus (PaLM, **B**), ventromedial hypothalamus (VMH, **C**) and the caudal region of the arcuate nucleus (cARC, **D**). (**E**) Histogram depicting the mean ± SEM percentage of total mCherry-labelled cells per hypothalamic nuclei in male and female mice. KNDy neurons in female mice receive significantly higher presynaptic input from the PeN, VMH (Ei) and cARC (Eiii) compared to males, but significantly lower input from the PaLM (Eii). Students t-test. Scale bars = 125 um. *p < 0.05. 3 V = third ventricle, ot = optic tract, VMPO = ventromedial preoptic nucleus, OVLT = organum vasculosum of the laminar terminalis, MPON = medial preoptic nucleus, POA = preoptic area, AVPV = anteroventral periventricular nucleus, LHA = lateral hypothalamus anterior, SON = supraoptic nucleus, ASO = accessory groups of the supraoptic nucleus, AHA = anterior hypothalamic area, DMH = dorsomedial hypothalamus, LH = lateral hypothalamus, PH = posterior hypothalamus, PMV = ventral premammilliary nucleus, Tu = tuberal nucleus, SuMM = supramammilary nucleus, PaV = PVN; ventral part, PaMP = PVN; parvicellular part.

The periventricular nucleus (PeN, Fig. 5A) and ventromedial hypothalamus (VMH, Fig. 5C) contained a significantly higher percentage of total afferent input to KNDy neurons in female mice compared to males (p < 0.05, Fig. 5E). The anteroventral periventricular nucleus (AVPV) also contained a higher percentage of total afferents in female mice, although this did not reach significance (Supplementary Table 2). Sex differences were not apparent when comparing the total PVN, however, the lateral magnocellular division of the PVN contained a significantly higher percentage of afferent input to KNDy cells in males compared to females (p < 0.01, Fig. 5B,Eii), indicating subdivision-specific sexual differentiation. Lastly, the percentage of afferents in the caudal subdivision of the ARC that were mCherry-positive and GFP-negative, and therefore presumably non-KNDy neurons, was significantly higher in female mice compared to males (p < 0.05, Fig. 5D,Eiii).

### Delineating the phenotype of afferent populations to KNDy neurons in male and female mice

The findings above demonstrate that significant differences in synaptic input to KNDy neurons occurs between sexes. To aid in identifying the phenotype of these populations, male (n = 6) mice and female (n = 4) mice in metestrus or diestrus were perfused 5 days post-RVDG transfection to minimize cell exposure to RVDG, which has been reported to produce cytotoxicity^49^. ERα was immunolabelled to delineate neuronal phenotypes within steroid-hormone sensitive populations, and confocal microscopy used to analyze ERα colocalization with mCherry in estrogen-sensitive hypothalamic nuclei. Initially, the number of ERα-positive cells in the ARC was compared in tissue collected 5 and 7 days post-RVDG transfection to compare whether a reduction in RVDG-transfection period improves immunolabeling for proteins. In both male and female mice, the number of ERα-positive cells was significantly higher 5 days post-RVDG transfection compared to 7 days post-RVDG transfection (Supplementary Fig. 4), supporting reduced cytotoxicity 5 days post-RVDG transfection.

**Figure 6:**
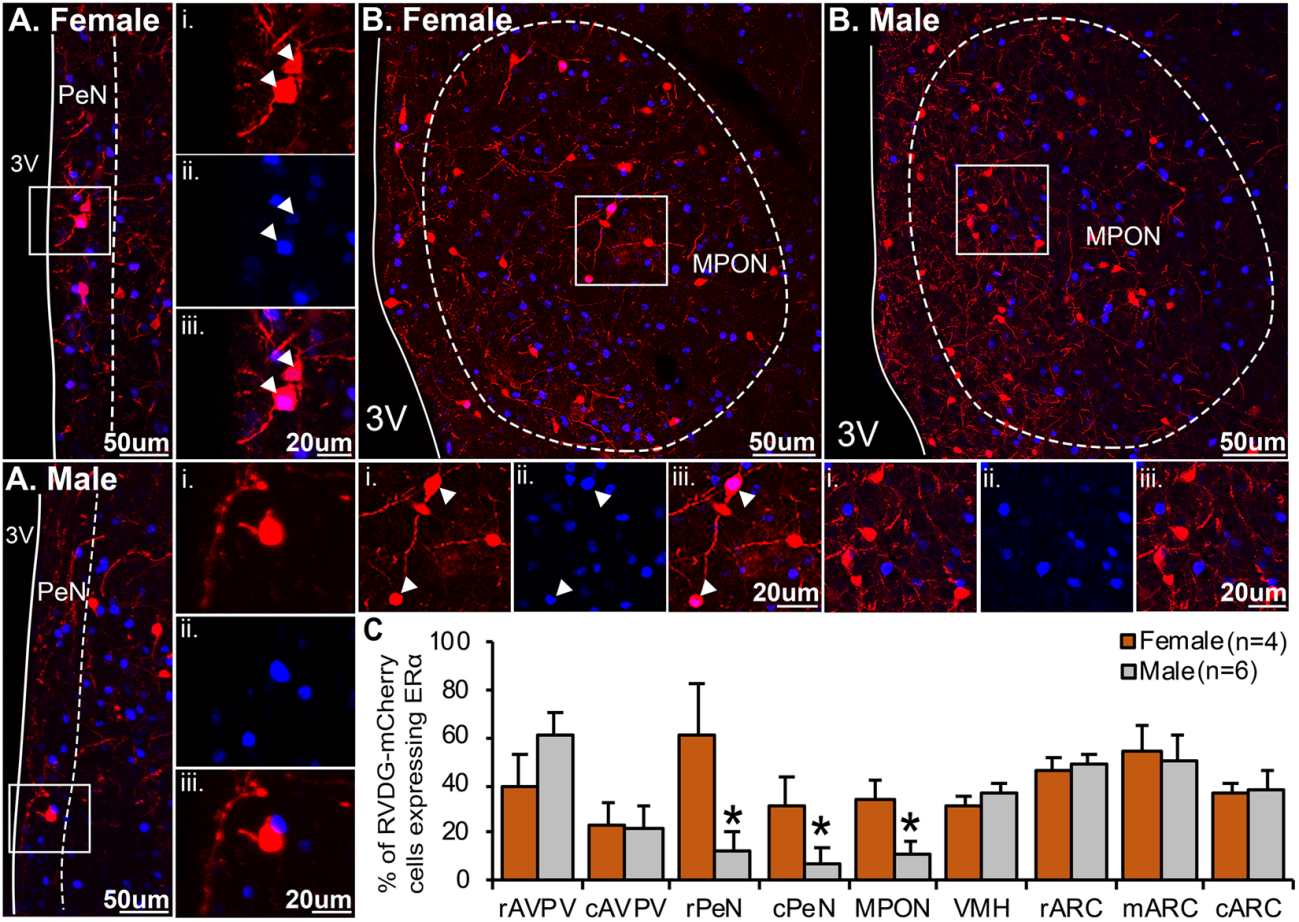
Sexually differentiated input to KNDy neurons from estrogen-sensitive cells in the periventricular nucleus and the medial preoptic area. Confocal images of mCherry-immunoreactive (ir) (red) afferent populations to KNDy neurons and estrogen receptor alpha (ERα)-positive nuclei (blue) in the periventricular nucleus (PeN, **A**) and medial preoptic nucleus (MPON, **B**) of male and female mice. High magnification images of mCherry-positive neurons (i), ERα positive nuclei (ii) and merged images from corresponding boxes in (**A**,**B**). (**C**) Histogram depicting the mean ± SEM percentage of afferent populations to KNDy mCherry-ir cells colocalized with ERα in the rostral (rAVPV) and caudal (AVPV) anteroventral periventricular nucleus, rostral (rPeN) and caudal (cPeN) PeN, ventromedial hypothalamus (VMH), and rostral (rARC), middle (mARC) or caudal (cARC) arcuate nucleus. The percentage of afferents to KNDy mCherry-ir cells colocalized with ERα is significantly higher in female mice compared to male mice in the rPeN, cPeN and MPON. No significant difference is detected in the rAVPV, cAVPV, VMH, rARC, mARC or cARC. 3 V, third ventricle. Student’s t-test.

**Figure 7:**
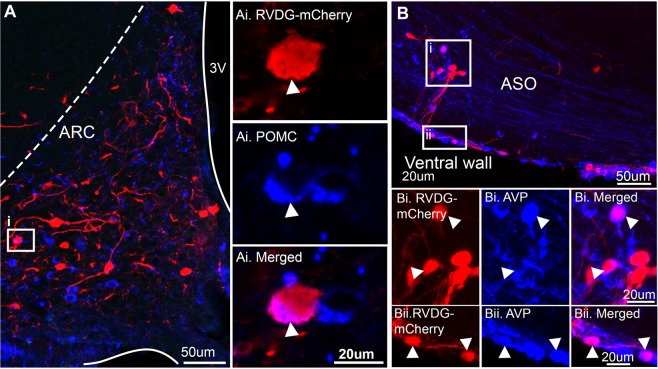
The peptidergic phenotype of afferents to KNDy subpopulations. Representative confocal images of immunoreactivity for RVDG-mCherry colocalized with either POMC in the arcuate nucleus (ARC, **A**) or arginine vasopressin (AVP) within the accessory group of the supraoptic nucleus (ASO, **B**). (i–ii) High magnification images of immunoreactivity correlating with insets in (**A**,**B**) with arrows indicating RVDG-mCherry co-expression with neuropeptides.

In this tissue, the percentage of RVDG-mCherry nuclei positive for ERα was significantly greater in female mice compared to males in the PeN (Fig. 6A, p < 0.05) and the MPON (Fig. 6B, p < 0.05). No significant differences were detected in the AVPV, VMH or ARC (Fig. 6C). Due to the large source of afferent inputs to KNDy neurons originating within the ARC, immunolabelling for POMC, a neuropeptide implicated in the metabolic regulation of reproduction, was performed. mCherry cells were colocalized with POMC immunolabelling in 2 out of 4 female mice and 3 out of 6 male mice (Fig. 7A). As a large percentage of afferent input to KNDy neurons was identified within the PVN, supraoptic nucleus (SON) and accessory groups of the supra optic nucleus (ASO), immunolabelling for common neuropeptides within these regions, oxytocin (OXT) and arginine vasopressin (AVP), was performed. Neither AVP nor OXT were colocalized with mCherry-ir neurons in the PVN. However, AVP was present in mCherry cells located within the ASO, both in a line of cells located medial to the optic tract (Fig. 7Bi) and along the ventral wall (Fig. 7Bii) in 4 out of 6 male mice and 2 out of 4 female mice.

## Discussion

This study suggests that KNDy neurons receive direct synaptic input from a remarkably diverse number of afferent populations throughout the brain, which may form the anatomical basis for the control of gonadotropin secretion over different reproductive and non-reproductive states. By using cell-specific retrograde monosynaptic rabies-mediated tract-tracing combined with 3D imaging of the afferent KNDy network in the transparent intact brain and detailed quantification in sectioned tissue, we determined that 90% of afferent input was located within hypothalamic nuclei, including regions that are essential for neuroendocrine processes that alter gonadotropin release. This strongly supports that KNDy cells act as a central nodal point to integrate external and internal signals that alter pulsatile gonadotropin secretion. Afferent populations to KNDy neurons were identified in hypothalamic and non-hypothalamic nuclei not previously associated with gonadotropin release, potentially indicating unexplored neuroendocrine functions or involvement of KNDy cells in non-neuroendocrine processes. Although viral-mediated tract-tracing of afferent neuronal circuits to KNDy cells has recently been described using the pseudorabies virus in male and female mice^50^, it is difficult to distinguish monosynaptic from polysynaptic circuits using this technique. This study, like ours, included the use of rabies-mediated tracing to discriminate monosynaptic input, however this was only carried out in a cohort of male mice and identified a subset of afferent populations described in the present study. Additional afferent populations identified here were located within the septal nucleus, bed nucleus of the stria terminalis, organum vasculosum of the lamina terminalis, lateral and medial preoptic area, anterior hypothalamic area, accessory groups of the supraoptic nucleus, ventromedial hypothalamus, dorsomedial hypothalamus, lateral hypothalamic area, posterior hypothalamic area, supramammillary nucleus, zona incerta, amygadalohippocampal area and subiculum. Therefore, the present study adds to our knowledge of brain nuclei with direct synaptic input, and likely the greatest modulatory impact, on KNDy neurons in both male and female mice. Importantly, we also revealed sexually-dimorphic afferent input to KNDy cells originating from regions involved in sexual behavior and the female-only preovulatory GnRH/LH surge, providing potential pathways through which sex-specific gonadotropin release is generated.

Although the rabies virus provides an unmatched ability to confirm monosynaptic input from large numbers of afferent populations, technical challenges need to be considered. The AAV and RVDG vectors used in this study were highly specific to Cre, however, due to the reciprocal connections between KNDy cells^9^, it is difficult to determine the number and location of initially transfected “starter” KNDy cells. To combat this, afferent populations were reported as the percentage of total mCherry transfected cells throughout the brain. AAV-TVA/GFP and RVDG-mCherry co-labeled cells were present in the contralateral ARC, which is potentially due to spread of viral vectors through the median eminence. However, it is likely that the majority of RVDG-mCherry-transfected cells represent contralateral KNDy connections, opposed to starter cells, as mCherry expression in the contralateral brain outside of the ARC was limited. This is in agreement with previous anatomical studies that identified projections from KNDy cells to the contralateral ARC and recent optogenetic studies^51–53^. Interestingly, only half of AAV-TVA/GFP transfected cells in the ipsilateral ARC expressed RVDG-mCherry, signifying more limited reciprocal connections between KNDy cells than initially expected^9^. This may denote cell groups or subpopulations of KNDy cells are interconnected, but extensive connections do not link the wider population.

A second consideration is the inherent toxicity that is displayed by the rabies virus, which limits the ability to label for proteins with high turnover rates. In this study, we found a reduced ability to label for steroid hormone receptors 7 days post-RVDG transfection. To address this, we performed a separate study by perfusing mice 5 days post-RVDG injection, permitting transfection in the same areas as reported with 7 days and limiting, although not eliminating, toxicity within the cell. This allowed confirmation of synaptic input from estrogen-sensitive neurons and specific neuropeptides but did not permit quantification of neuropeptide colocalization. New generations of rabies vectors are being developed that prevent toxicity, either through a self-inactivation strategy^54^ or by removing the ability of the virus to replicate through deletion of the viral polymerase gene^55^. These vectors have been used to study synaptic input in region- or projection-specific neurons. However, they have a limited ability to be used in Cre recombinase mouse lines, and therefore, targeting to cells that are defined by neuropeptidergic phenotype may be difficult. Alternate rabies virus strains have also been shown to prolong the survival of neurons^56^, however the toxicity is not overcome completely and it is unclear if neuropeptide levels can be retained.

Brain-wide tracing determined that the largest source of input within the hypothalamus arose from local non-KNDy cells, supporting that KNDy neurons receive major regulatory input from ARC cells that relay metabolic signals^42,45^. This finding is consistent with recent studies testing individual presynaptic pathways to KNDy cells using optogenetic and chemogenetic techniques. The stimulation of leptin-sensitive ARC neurons expressing GABA and agouti-related peptide (AgRP) directly inhibits KNDy neuron activity and suppresses fertility in mice, indicating direct synaptic input that relays starvation signals to suppress gonadotrophin secretion^45^. Further, proopiomelanocortin (POMC) cleavage products, cocaine- and amphetamine-regulated transcript and alpha-melanocyte stimulating hormone, directly stimulate KNDy neurons^42,57^, which is in line with our findings. Interestingly, the deletion of ERα from POMC cells impairs estradiol negative feedback and fertility in female mice^58^, indicating POMC may be recruited following puberty to relay estradiol negative feedback information to KNDy cells.

Although no afferent populations to KNDy cells were present in one sex while absent in the other, multiple hypothalamic nuclei contained sexually-dimorphic afferent input to KNDy cells that may underlie sex-dependent changes in LH pulse frequency and amplitude. A higher percentage of afferents to KNDy cells were detected in the RP3V of females compared to males, specifically within the PeN. Further, of the afferent cells detected in the PeN, a significantly greater percentage co-expressed ERα in female mice compared to males. The RP3V contains multiple estrogen-sensitive populations^59^, including a greater estrogen-sensitive rostral kisspeptin population (RP3V kisspeptin neurons) in females compared to males that is implicated in mediating the preovulatory surge in GnRH/LH release in response to high estradiol levels in females. RP3V kisspeptin neurons directly synapse onto GnRH neurons that are activated during the LH surge^60–63^. Although this is strong support that RP3V kisspeptin cells directly stimulate GnRH neuron activity during surge secretion, KNDy neurons may also play a role in modulating the surge. In the sheep and primate, kisspeptin expression in KNDy neurons is elevated and a proportion of KNDy neurons express cFos during the LH surge^64–66^, suggesting that a subpopulation of KNDy neurons are involved in surge generation. Further, ablation of KNDy neurons in the rat brain amplifies the magnitude of the LH surge, likely through a lack of dynorphin inhibition of RP3V kisspeptin neurons^67,68^. Intriguingly, although the total RVDG-mCherry afferents in the AVPV trended towards a female-dominant sex difference, no difference in the percentage of estrogen-sensitive afferents to KNDy cells was detected within the AVPV, despite the presence of kisspeptin cells in this region^59^. It is possible that the AVPV and PeN form functionally distinct populations, however, there is little evidence for this to date. Alternatively, it is possible that a sex-difference was masked by other estrogen-sensitive cell types present in the AVPV^59^.

KNDy neurons in female mice received significantly higher input from the VMH compared to males, and, a higher percentage of input from the MPON contain ERα. A well characterized estrogen-sensitive circuit between the ARC, MPON and VMH is essential for the expression of lordosis behavior in female rodents driven by high estradiol levels required for the preovulatory LH surge^69,70^. The identification of sexually dimorphic input from the VMH and MPON to KNDy cells implies potential integration of KNDy cells in circuits regulating sex behavior, although the functional significance of this connection will need to be explored. Potentially, VMH/MPON input to KNDy cells provides a pathway through which sexual stimulation in rodents induces cFos expression within GnRH neurons^71,72^.

Unexpected sexually-dimorphic inputs to KNDy neurons were detected from the caudal region of the ARC, with females demonstrating robustly higher afferent input compared to males in this subdivision. Although a recent Drop-seq analysis of heterogeneity in the ARC detected over 50 transcriptionally unique cell populations^43^, the regional and functional organization of these subpopulations in the rostral-caudal extent of the ARC is largely unknown. Differences in the responses of caudal ARC KNDy neurons to AVP and vasoactive intestinal peptide stimulation have been recorded in the mouse^73^, and, cFos is differentially expressed in caudal ARC KNDy neurons in the sheep following treatment with a somatostatin receptor antagonist^74^. However, the phenotype of caudal ARC cells providing sex- and region-specific presynaptic contact to KNDy cells need further study. In the ARC, approximately half of afferent inputs to KNDy cells expressed ERα, supporting multiple neuronal phenotypes provide synaptic contact with KNDy cells. However, no significant difference in the percentage of afferent neurons containing ERα was detected between males and females.

A greater percentage of afferents to KNDy cells in the lateral magnocellular region of the PVN was the only male-dominant sex difference detected. Cells in this subdivision are typically described to contain oxytocin and vasopressin, and interestingly, a population of oxytocin neurons here project to the spinal cord to control ejaculation reflex^75,76^. Although colocalization with RVDG-mCherry cells and oxytocin was not detected in this study, this may have been due to technical difficulties in retaining neuropeptide expression. Immunolabeling in this study only detected colocalization of rabies-transfected cells with neuropeptides in some animals per group, indicating future work comparing synaptic input from specific neuropeptidergic populations to KNDy cells will require supplemental techniques in addition to the rabies virus. Of note, AVP was detected in a striking population of afferent cells located within the accessory groups of the supraoptic nucleus. During development of the SON, cells migrate ventrolaterally towards the outer border of the optic chiasm. The more ventrally situated cells observed here are likely the result of arrested lateral migration^77^ and contain the same neuropeptidergic phenotype as the SON. In agreement with this, AVP-immunoreactivity was colocalized with cells in the ASO, specifically, along the ventral wall and medial to the optic chiasm.

In contrast to other brain areas, mCherry-positive afferents in the brainstem and midbrain were only detected within a portion of the male and female animals studied. This was a surprising result given the important role of brainstem catecholamine-producing neurons in gonadotropin release, such as norepinephrine^78–83^. Of note, input to KNDy neurons was detected from the male and female dorsal raphe nucleus and female locus coeruleus which are major sources of serotonin and norepinephrine, respectively, and also provide direct synaptic input to GnRH neurons^84^. Both are small nuclei that exert powerful modulatory effects on neurotransmission in many brain regions^85–88^. Therefore, a minor input may have significant and physiological effects on KNDy neuron activity. Alternatively, brainstem modulation of gonadotrophin release may take place primarily at GnRH neurons^84,89–92^.

In conclusion, this study provides unequivocal identification of cell populations with primary synaptic input to KNDy neurons, providing a critical framework that can be used in future studies identifying the functional impact of afferent populations to KNDy neurons. Importantly, the ability to distinguish the complete array of upstream neuronal populations modulating KNDy neuron activity in response to peripheral cues provides new opportunities to manipulate gonadotropin release. Currently, drugs targeting KNDy peptides are being developed to treat women of reproductive age with hypothalamic amenorrhea^93–96^ and women with post-menopausal hot flushes^97,98^. Identifying upstream regulators of the KNDy neuronal network may therefore help isolate neuronal targets for therapeutic agents to both regulate fertility and to prevent fertility disorders, such as those resulting from steroid hormone feedback impairments or metabolic suppression of fertility.

## Materials and methods

### Animals

Male and female Kiss1-Cre mice, in which Cre-recombinase expression is driven by *Kiss1-*Cre regulatory elements^99^ (Breeding pairs donated by Dr. Carol Elias, JAX mice, stock #023426), and Kiss1-Cre mice crossed with tdTomato fluorescent reporter mice (B6.Cg-Gt-(ROSA)^26Sortm9(CAG-tdTomato)Hze/J^, JAX mice, stock #007907) were bred and housed in the University of Mississippi Medical Center animal facility on a 12 hour light/dark cycle and given access to food and water *ad libitum*. Kiss1-Cre is a transgenic mouse line with Cre-recombinase expression driven by *Kiss1* regulatory elements^99^. Experimental procedures in mice were conducted from 50 days of age. All experimental protocols and procedures were approved by the University of Mississippi Medical Center Institutional Animal Care and Use Committee and conform to guidelines outlined by the United States National Institutes of Health for animal research.

### Viral vectors

Adenoassociated virus’s (AAVs) AAV8-EF1a-FLEX-GT (AAV-TVA/GFP, 1.86E^+12^ pfu) and AAV8-CAG-FLEX-oG-WPRE-SV40-PA (AAV-oG, 8.91E + 13) and EnvA glycoprotein-Deleted Rabies-mCherry virus (RVDG, 3.78^E+07^) were prepared and purified by the Gene Transfer Targeting and Therapeutics Core at the Salk Institute of Biological Studies (La Jolla, CA).

### Stereotaxic viral injection

Adult Kiss1-Cre heterozygous male and female mice (Dr. Carol Elias, JAX #023426) and wildtype littermates (n = 5) were anesthetized with isoflurane (2%) and placed in a stereotaxic frame (Stoelting Co. IL, USA). A small hole was drilled into the skull 1 mm posterior to Bregma and 0.3 mm lateral to the midline. A 29-gauge cannula containing 25 nL of AAV-TVA-GFP and 75 nL of AAV-oG (total 100 nL volume) was injected 5.9 mm ventral to dura in the unilateral ARC using a Hamilton syringe. Three weeks later, mice were again anesthetized and placed in a stereotaxic frame for the injection of RVDG (400 nL) in the same coordinates as above. Control mice received either AAV-TVA/GFP (n = 4) or AAV-oG (n = 4) followed by RVDG injections. Kiss1-Cre mice crossed with a tdTomato reporter line (JAX #007909) were injected as described above with AAV-TVA/GFP and AAV-oG (n = 3 males, n = 3 females, Fig. 1). Either 7 days (n = 5 females, n = 4 males; Figs 2, 4 and 5) or 5 days (n = 4 females, n = 6 males, Figs 6 and 7) following RVDG injection, mice were given an overdose of pentobarbital (3 mg/mL, intraperitoneal), vaginal cytology was collected from females to determine estrous cycle stage, and mice perfused transcardially with 4% paraformaldehyde (PFA). At the time of perfusion, all female mice used in this study were either in metestrus or diestrus. Brains were collected and post-fixed in PFA for 1 hour before overnight incubation in 20% sucrose in PBS. Male brains (n = 2) for optical tissue clearing were incubated at 4 C overnight in 4% PFA and stored in PBS with sodium azide.

### Immunofluorescence

Following sucrose immersion, brains from Kiss1-Cre and WT mice collected 7 days post-RVDG transfection, and, brains from Kiss1-Cre/tdTomato mice collected 3 weeks following AAV-TVA/GFP transfection were cut into 3 parallel series of coronal sections at 30 µm thickness using a freezing microtome (H400R, Micron, Germany). Brains from Kiss1-Cre mice collected five days post-RVDG transfection were cut into 5 parallel series of 25 µm thick coronal sections. To perform free-floating immunohistochemistry, all tissue was initially washed in 0.1 M PBS for a minimum of 4 hours, exposed to 1% H202 (10 min in 0.1 M PBS) and incubated for 1 hour in antibody incubation solution (0.1% bovine serum albumin (Thermo Fisher Scientific) and 0.4% Triton-X 100 in 0.1 M PBS).

For whole-brain mapping of viral transfection, every third section containing the prefrontal cortex (1.18 mm anterior to Bregma) through to the brainstem (7.5 mm posterior to Bregma) was immunolabeled to enhance endogenous GFP and mCherry. Tissue was incubated in rabbit antiserum against mCherry (1:4000, Abcam, Cat AB167453, RRID:AB_2571870) and chicken antiserum against GFP (Aves Laboratories, 1:2000, Cat GFP-1020, RRID:AB_10000240) in incubation solution for 17 hours at RT. Sections were washed in PBS and incubated in Dylight goat anti-chicken 488 and Dylight donkey anti-rabbit 550 (1:200) for 30 min before a final wash in 0.1 M PB. Kiss1-Cre/tdTomato control mice transfected with AAV-TVA-GFP were immunolabeled for GFP only using rabbit antiserum to GFP and Dylight goat anti-chicken 650 to eliminate bleed-through of tdTomato into the 488 range.

To identify steroid hormone-sensitive mCherry-labelled cells and colocalized neuropeptides, fluorescent immunohistochemistry was performed as previously described^100^. Briefly, sections were incubated overnight in incubation solution with either rabbit anti-ERα (1:40,000, Millipore, Cat AB1565 RRID: AB_310395), rabbit anti-AVP (1:400,000, Millipore, Cat AB1565, RRID: AB_90782), rabbit anti-oxytocin (1:80,000, Millipore, Cat AB911, RRID: AB_2157629) or rabbit anti-POMC (1:400,000, Phoenix Pharmaceuticals, Cat H029-30, RRID: AB_2307442). Sections were incubated with biotinylated goat anti-rabbit IgG (1:500 in incubation solution, 1 h; Vector Laboratories, Burlingame, CA, USA) followed by avidin-horseradish peroxidase complex (ABC-Elite; 1:500 in PBS, Vector). Next, sections were reacted with biotinylated tyramine /tissue sample amplification (TSA, 1:250 in PBS containing 1uL/mL 3% H202; NEL700/700 A, PerkinElmer Life Sciences, Boston, MA, USA) for 10 min, followed by Dylight 650 conjugated streptavidin (1:100 in PBS, 30 min; Pierce Biotech., Rockford, IL, USA). Sections were then incubated overnight with chicken anti-GFP and rabbit anti-mCherry antibodies as described above. Finally, sections were incubated with Dylight goat anti-chicken 488 and Dylight donkey anti-rabbit 555 for 30 min in incubation solution. Sections were mounted on superfrost charged glass slides (Thermo Fisher Scientific, Cat# 12-550-20, Waltham, MA, USA), air dried and coverslipped using an aqueous mounting medium (Gelvatol^101^) containing the anti-fade agent 1,4-diazabicyclo(2,2)octane (Sigma-Aldrich; 50 mg/mL).

### Image analysis

To analyze co-expression of AAV-TVA/GFP with either Kiss1-Cre/tdTomato or RVDG-mCherry, immunolabelled sections containing the rostral, middle and caudal regions of the ARC were imaged using a laser-scanning Nikon D-Eclipse C1 confocal system (Nikon Corporation) attached to a Nikon Eclipse E800 microscope (Nikon Corporation) using a 20x objective with 1.5x zoom. Confocal Z-stacks of 1 µm thick optical sections were captured through the ARC. Two sections per rostral, middle and caudal region of the ARC were imaged per animal, and the percentage of tdTomato-positive cells colocalized with GFP and the percentage of GFP-positive cells colocalized with tdTomato or mCherry calculated.

Brain-wide mapping of RVDG-mCherry cells was conducted using epifluorescent microscopy (DM500B, Lecia Microsystems) and a digital camera (Microfire A/R; Optronics) paired with MicroBrightField Neurolucida Software (Williston, Vermont USA) to permit rapid imaging of brain series. For whole-brain mapping of afferents to KNDy neurons, cells positive for mCherry were quantified in two representative brain sections per nuclei per animal using Image J software. In the ARC, mCherry-labelled cells were separated into mCherry only and mCherry + GFP neurons. To normalize for differences in viral transfection, the number of mCherry-positive cells in each brain nuclei were expressed as a percentage of the total mCherry-positive cells quantified across the brain. Brain regions containing mCherry-positive cells in 100% of animals quantified are listed in Supplementary Tables 1 and 2. Excluding the periaqueductal grey, mCherry-positive cells in the midbrain and brainstem were only present in a portion of animals, as depicted in Supplementary Fig. 3 and was therefore excluded from quantitative analysis with other brain areas.

Rapid imaging using epifluorescent microscopy and Neurolucida software, as described above, was used to map mCherry co-expression with ERα or neuropeptides. Areas displaying regions of colocalization were quantified using confocal microscopy by imaging z-stacks of 1 µm thick optical sections using a Nikon D-Eclipse C1 laser-scanning confocal system (Nikon Corporation) attached to a Nikon Eclipse E800 microscope (Nikon Corporation). Confocal images using a 20x or 60x objective were obtained to confirm co-expression of mCherry with ERα in the rostral and caudal AVPV, rostral and caudal PeN, MPON, VMH and rostral, middle and caudal ARC using a 20x objective. In OXT- and AVP-labeled sections, confocal images were obtained in the paraventricular nucleus, supraoptic nucleus and accessory group of the supraoptic nucleus (ASO) using a 20x objective. In POMC-labeled brain sections, confocal images of co-expression were obtained in the rostral, middle and caudal ARC using a 20x and 60x objective with oil immersion.

In all analyses, brain regions were determined by anatomical landmarks and based on the Mouse Brain Atlas in Stereotaxic Coordinates by Franklin and Paxinos, second edition^102^.

### Statistical analysis

Two-way ANOVAs with Tukey post-hoc tests were used to analyze co-expression of AAV-TVA/GFP-transfected neurons and RVDG-mCherry in the rostral-caudal ARC of male and female mice. All other comparisons in male and female mice was performed using two-tailed Student’s T Tests. In all analyses, experimenters were blinded to experimental group and statistical comparisons were made using SigmaPlot Software.

### iDISCO+-mediated wholemount immunolabelling and optical tissue clearing

Intact mouse brains transfected with AAV and RVDG viruses (n = 2, male mice; Fig. 3, Video 1) were immunolabelled and rendered transparent as previously described^103^. Briefly, intact mouse brains were dehydrated in increasing concentrations of methanol, bleached with 5% hydrogen peroxide overnight at 4 C and rehydrated in decreasing concentrations of methanol. Brains were blocked in a solution containing normal goat serum for 2 days at 37 C on a shaker, followed by incubation in rabbit anti-mCherry (Abcam, 1:1000) and chicken anti-GFP (Aves Laboratories, 1:1000) primary antisera on a rotating shaker at 37 C for 7 days. Brains were next incubated at 37 C on a rotating shaker for 7 days in goat anti-rabbit 555 (1:400) and goat anti-chicken 647 (1:400) secondary antisera. Following immunolabelling, brains were dehydrated a final time using increasing concentrations of methanol. Next, brains were delipidated using rotation in a 33% dichloromethane/66% methanol solution for 3 hours, washed twice in 100% dichloromethane for 15 minutes and rendered completely transparent using overnight incubation in dibenzyl ether (DBE). Brains were submerged in a chamber containing DBE and imaged using bidirectional light sheet microscopy (Ultramicroscope I, LaVision Biotec) with a 2x/0.5NA objective (MVPLAPO Olympus). Stacks of TIFF images throughout the whole mouse brain were collected using a sCMOS camera (Andor Neo) and ImSpectorPro software (LaVision BioTec) at 1x magnification with a 2μm optical interval. Mosaic stacks of 16-TIFF images were converted to IMARIS files (.ims, Bitplane) and 3D projections of z-stack images were generated using the volume rendering function. In files generated using 1x magnification, IMARIS segmentation tools were using to individually render and pseudocolor brain region-specific mCherry-labelled cells. Three-dimensional images and movies were generated using the ‘snapshot’ and ‘animation’ tools in IMARIS, and video files were edited using Adobe Premier Pro CC v.13.0.2.

## Supplementary information

Video 1.

Supplementary information

## Data Availability

The datasets generated and analyzed during the current study are available from the corresponding author upon request.
