# Supplementary material for: Kisspeptin/Neurokinin B/Dynorphin (KNDy) cells as integrators of diverse internal and external cues: evidence from viral-based monosynaptic tract-tracing in mice
Source: Sci Rep. 2019 Oct 14;9:14768. doi: 10.1038/s41598-019-51201-0 (PMC6791851; doi:10.1038/s41598-019-51201-0)
Supplement: Supplementary file 2 — Supplementary information [file 41598_2019_51201_MOESM2_ESM.pdf]

Kisspeptin/Neurokinin B/Dynorphin (KNDy) cells as integrators of diverse internal and external cues: evidence from viral-based monosynaptic tract-tracing in mice

Aleisha M. Moore*, Lique M. Coolen, Michael N. Lehman

Brain Health Research Institute and Dept. of Biological Sciences, Kent State University, Kent, OH

List of abbreviations:

3V–third ventricle; AHA–anterior hypothalamic area; AGRP-agouti-related peptide; ASO-accessory groups of the supra optic nucleus; ARC-arcuate nucleus; APVP-anteroventral periventricular nucleus; AVP-arginine vasopressin; BNST-bed nucleus of the stria terminalis; CART-cocaine- and amphetamine-regulated transcript; CRH-corticotropin-releasing hormone; DMH-dorsomedial hypothalamus; ERα-estrogen receptor alpha; f–fornix; GnRH-gonadotropin-releasing hormone; KNDy-Kisspeptin, Neurokinin B, Dynorphin; LA–lateroanterior hypothalamus, LH – luteinizing hormone; LHA–lateral hypothalamus; LPO–lateral preoptic area; MPA–medial preoptic area; MPON-medial preoptic nucleus; NPY-neuropeptide Y; ot-optic tract; OVLT-organum vasculosum of the laminar terminalis; OXT-oxytocin; PeN-periventricular nucleus; PFA-paraformaldehyde; PH-posterior hypothalamus; PMV-ventral premammillary nucleus; PVN-paraventricular nucleus; POMC-proopiomelanocortin; PACAP-pituitary adenylate cyclase-activating peptide, RVDG-rabies glycoprotein-deleted virus, SCh-suprachiasmatic nucleus, SON-supraoptic nucleus; SuMM-supramammilary nucleus; Tu-tuberal nucleus; VMH-ventromedial hypothalamus; VMPO-ventromedial preoptic nucleus.

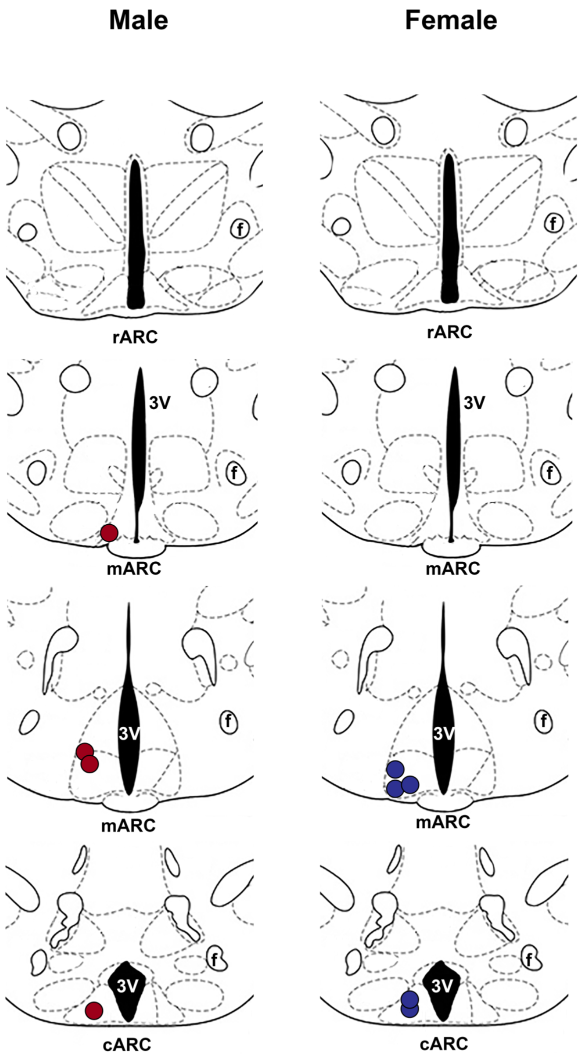

Supplementary Figure 1. Map of RVDG-mCherry injection sites.
Schematic diagrams adapted from the Paxinos and Franklin Mouse Brain atlas [102] illustrating injection sites within the middle (mARC) and caudal (cARC) regions of the arcuate nucleus in male (red dot/animal) and female (blue dot/animal) mice that underwent 7 days of RVDG transfection.

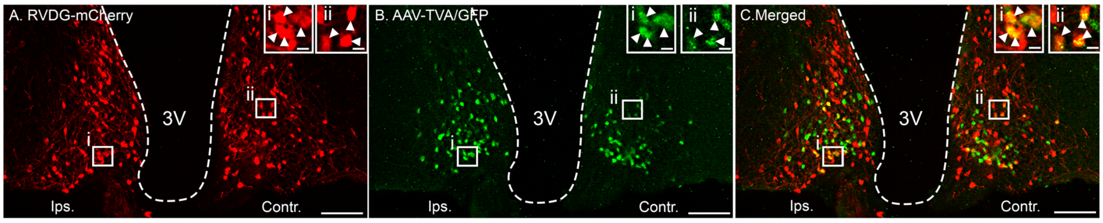

Supplementary Figure 2. Expression of viral vectors in the contralateral arcuate nucleus.

Representative epifluorescent images of the arcuate nucleus on the ipsilateral (Ips.) and contralateral (Contr.) side of viral vector injection containing RVDG-mCherry-immunoreactive (-ir) cells (A), AAV-TVA/GFP-ir cells (B) and merged images (C), bars=100um. i-ii) Insets illustrating mCherry+GFP colocalized cells (white arrows) in the ipsilateral (i) and contralateral (ii) ARC. Bars=10um.

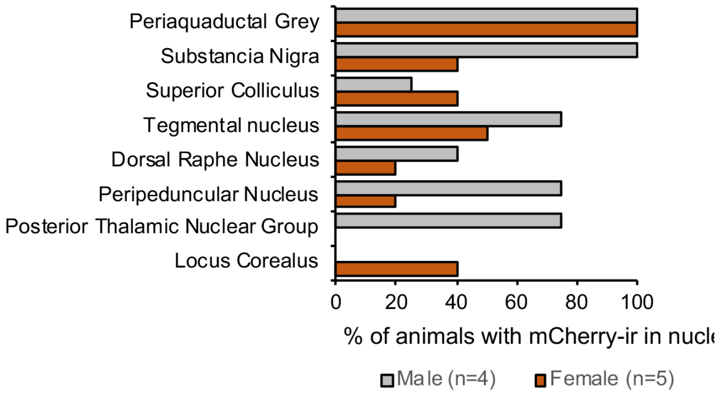

Supplementary Figure 3. Variable mCherry-labelled presynaptic cells identified in the midbrain and brainstem of male and female mice.
Histogram showing the percentage of female (orange bars) and male (grey bars) with RVDG-mCherry immunoreactivity in midbrain and brain-stem nuclei.

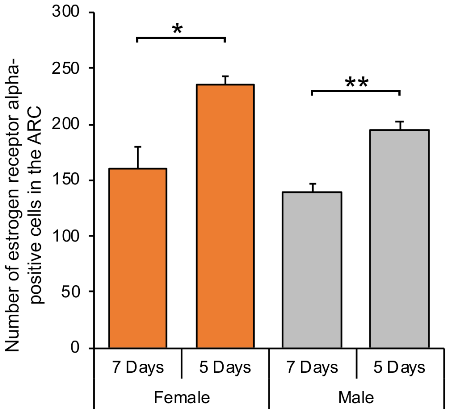

Supplementary Figure 4. Estrogen receptor alpha expression is reduced 7 days post-RVDG transfection compared to 5 days post-RVDG transfection

Histogram showing the mean ± SEM number of estrogen receptor alpha-positive nuclei within the ipsilateral arcuate nucleus of male and female mice at 7- and 5-days post-transfection with RVDG. * p<0.05, ** p<0.01 student's t-test.

Supplementary Table 1. Mean ± SEM number of RVDG-mCherry cells in brain nuclei of male and female mice.

Brain region	Nucleus	Division	Female	Male	P-value
Septal nucleus	Septal nucleus	Lateral part	8.0 ± 1.7	6.1 ± 1.0	0.4
		Medial part	2.61 ± 2.7	3.1 ± 0.7	0.88
Basal forebrain	Bed nucleus of the stria terminalis	Medial division, anterior part	4.8 ± 2.8	5.1 ± 0.7	0.93
		Lateral division, ventral part	1.0 ± 0.6	5.8 ± 2.2	0.052
		Medial division, posterolateral part	5.3 ± 2.3	16.8 ± 13.2	0.37
		Medial division, posterointermediate part	13.3 ± 5.3	11.1 ± 3.9	0.76
Preoptic area and Hypothalamus	Ventromedial preoptic nucleus		5.6 ± 2.2	4.5 ± 1.2	0.7
	Organum vasculosum of the lamina terminalis		0.8 ± 0.2	2.5 ± 0.6	*0.02*
	Medial preoptic nucleus	Medial part	23.3 ± 6.3	37.8 ± 6.1	0.15
		Lateral part	8.8 ± 2.4	15.8 ± 2.3	0.08
	Preoptic area	Lateral part	4.6 ± 1.9	2.9 ± 1.5	0.52
		Medial Part	9.4 ± 2.1	6.8 ± 1.2	0.35
	Anteroventral periventricular nucleus	Rostral	20.5 ± 5.9	15.0 ± 5.1	0.51
		Caudal	25.0 ± 6.6	20.9 ± 5.7	0.66
	Periventricular nucleus	Rostral	5.5 ± 1.6	2.1 ± 1.0	0.14
		Caudal	6.5 ± 2.1	2.3 ± 0.3	0.12
	Paraventricular nucleus	Ventral part	16.5 ± 7.2	18 ± 4.4	0.87
		Anterior parvicellular part	7.5 ± 1.8	14.3 ± 1.2	*0.02*
		Lateral magnocellular part	15.2 ± 6.9	40.6 ± 8.0	*0.048*
		Medial magnocellular part	19.5 ± 7.4	17.0 ± 3.9	0.598
		Medial parvicellular part	2.5 ± 1.2	2.5 ± 1.2	1
	Lateroanterior hypothalamic nucleus		5.4 ± 2.2	9.9 ± 1.0	0.13
	Anterior hypothalamic area	Central Part	5.7 ± 1.3	13.5 ± 3.6	0.06
		Anterior Part	5.3 ± 1.1	17.1 ± 2.7	*0.003*
		Posterior Part	7.6 ± 2.6	12.8 ± 2.8	0.22
	Ventromedial Hypothalamus	Ventral part	11.9 ± 3.6	13.3 ± 0.1	0.74
		Central part	18.5 ± 2.5	13.4 ± 1.6	0.15
		Dorsal part	9.2 ± 1.1	8.4 ± 1.5	0.67
	Supraoptic nucelus	Supraoptic nucleus	10.9 ± 3.0	12.1 ± 1.2	0.75
		Accessory groups of the supraoptic nucleus	15.6 ± 4.5	38.3 ± 12.6	0.10
	Arcuate nucleus	Rostral	45.8 ± 7.6	47.7 ± 12.6	0.9
		Middle	50.8 ± 8.5	88.5 ± 23.7	0.14
		Caudal	93.5 ± 24.2	64.9 ± 15.4	0.38
	Dorsomedial hypothalamic nucleus	Compart part	11.5 ± 2.9	11.4 ± 2.4	0.98
		Ventral Part	11.8 ± 3.7	14.3 ± 3.2	0.636
		Dorsal Part	16.6 ± 3.6	15.0 ± 3.1	0.75
	Lateral hypothalamic area		5.4 ± 1.4	16.3 ± 5.2	0.06

	Posterior hypothalamic area		4.6 ± 1.0	5.9 ± 2.2	0.58
	Premammillary nucleus	Ventral Part	20.4 ± 6.1	39.8 ± 12.1	0.17
		Dorsal Part	4.2 ± 3.0	7.3 ± 4.3	0.56
	Medial tuberal nucleus		7.5 ± 1.1	16.0 ± 2.0	*0.006*
	Supramammilary nucleus		2.4 ± 0.7	3.0 ± 1.1	0.65
Subthalamus	Zona incerta		2.0 ± 0.9	1.8 ± 0.6	0.33
Thalamus	Paraventricular thalamic nucleus	Anterior Part	3.4 ± 0.8	3.5 ± 1.3	0.95
Amygdala	Medial amygdala	Anterior Dorsal Part	1.3 ± 0.3	2.5 ± 0.5	0.07
		Posterodorsal Part	1.7 ± 0.4	1.5 ± 0.3	0.72
	Amygdalohippocampal area	Posteromedial Part	2.0 ± 0.8	3.9 ± 1.4	0.25
Hippocampal formation	Subiculum		4.8 ± 1.4	5.0 ± 1.2	0.92
Midbrain	Periaquaductal Grey		1.8 ± 0.2	2.9 ± 1.3	0.38

Supplementary Table 2. Mean ± SEM percentage of total RVDG-mCherry cells in brain nuclei of male and female mice.

Brain region	Nucleus	Division	Female (%)	Male (%)	P-value
Septal nucleus	Septal nucleus	Lateral part	1.34 ± 0.22	0.82 ± 0.06	0.085
		Medial part	0.41 ± 0.14	0.44 ± 0.11	0.877
Basal forebrain	Bed nucleus of the stria terminalis	Medial division, anterior part	0.68 ± 0.27	0.70 ± 0.08	0.952
		Lateral division, ventral part	0.22 ± 0.14	0.89 ± 0.36	0.098
		Medial division, posterolateral part	0.76 ± 0.26	2.41 ± 1.98	0.382
		Medial division, posterointermediate part	1.89 ± 0.5	1.46 ± 0.45	0.545
Preoptic area and Hypothalamus	Ventromedial preoptic nucleus		0.90 ± 0.21	0.61 ± 0.16	0.340
	Organum vasculosum of the lamina terminalis		0.15 ± 0.05	0.35 ± 0.1	0.103
	Medial preoptic nucleus	Medial part	3.82 ± 0.35	5.29 ± 1.1	0.203
		Lateral part	1.52 ± 0.27	2.12 ± 0.14	0.114
	Preoptic area	Lateral part	0.73 ± 0.21	0.37 ± 0.17	0.230
		Medial Part	1.81 ± 0.48	0.91 ± 0.11	0.146
	Anteroventral periventricular nucleus	Rostral	3.24 ± 0.38	1.93 ± 0.43	0.057
		Caudal	4.00 ± 0.56	2.71 ± 0.57	0.156
	Periventricular nucleus	Rostral	0.86 ± 0.19	0.31 ± 0.15	0.062
		Caudal	0.98 ± 0.19	0.30 ± 0.02	0.017
	Paraventricular nucleus	Ventral part	2.41 ± 0.63	2.65 ± 0.85	0.823
		Anterior parvicellular part	1.30 ± 0.2	1.96 ± 0.12	0.034
		Lateral magnocellular part	2.30 ± 0.63	5.78 ± 1.42	0.045
		Medial magnocellular part	3.04 ± 0.81	2.46 ± 0.72	0.598
		Medial parvicellular part	0.37 ± 0.1	0.31 ± 0.11	0.704
	Lateroanterior hypothalamic nucleus		0.86 ± 0.19	1.35 ± 0.1	0.073
	Anterior hypothalamic area	Central Part	1.08 ± 0.3	1.88 ± 0.56	0.228
		Anterior Part	1.12 ± 0.44	2.36 ± 0.38	0.079
		Posterior Part	1.22 ± 0.24	1.83 ± 0.48	0.264
	Ventromedial Hypothalamus	Ventral part	1.95 ± 0.29	1.86 ± 0.18	0.885
		Central part	3.47 ± 0.7	1.84 ± 0.22	0.087
		Dorsal part	1.77 ± 0.28	1.12 ± 0.11	0.088
	Supraoptic nucelus	Supraoptic nucleus	2.07 ± 0.63	1.73 ± 0.41	0.683
		Accessory groups of the supraoptic nucleus	3.20 ± 1.33	5.34 ± 1.91	0.373
	Arcuate nucleus	Rostral	7.92 ± 0.66	6.15 ± 0.62	0.188
		Middle	9.46 ± 1.59	11.70 ± 2.29	0.43
		Caudal	15.2 ± 1.85	8.47 ± 1.10	0.023
	Dorsomedial hypothalamic nucleus	Compart part	1.93 ± 0.52	1.54 ± 0.28	0.548
		Ventral Part	2.06 ± 0.19	1.89 ± 0.23	0.784
		Dorsal Part	2.83 ± 0.87	2.08 ± 0.5	0.240

	Lateral hypothalamic area		1.22 ± 0.41	2.15 ± 0.7	0.267
	Posterior hypothalamic area		0.84 ± 0.34	0.74 ± 0.18	0.817
	Premammillary nucleus	Ventral Part	3.63 ± 0.87	5.08 ± 1.06	0.328
		Dorsal Part	0.63 ± 0.41	0.93 ± 0.54	0.675
	Medial tuberal nucleus		1.37 ± 0.18	2.24 ± 0.35	0.051
	Supramammilary nucleus		0.43 ± 0.11	0.27 ± 0.16	0.423
Subthalamus	Zona incerta		0.53 ± 0.28	0.26 ± 0.11	0.443
Thalamus	Paraventricular thalamic nucleus	Anterior Part	0.50 ± 0.10	0.50 ± 0.2	1
Amygdala	Medial amygdala	Anterior Dorsal Part	0.20 ± 0.08	0.34 ± 0.04	0.193
		Posterodorsal Part	0.31 ± 0.04	0.20 ± 0.03	0.074
	Amygdalohippocampal area	Posteromedial Part	0.33 ± 0.09	0.49 ± 0.12	0.312
Hippocampal formation	Subiculum		0.54 ± 0.15	0.63 ± 0.13	0.674
Midbrain	Periaquaductal Grey		0.60 ± 0.18	0.24 ± 0.07	0.135

Video 1. Animated 3D rendering of afferent KNDy neurons in the transparent intact mouse brain using rabies-mediated tract tracing and optical tissue clearing

3D rendering using IMARIS software of presynaptic KNDy neurons in the intact mouse brain following rabies-mediated tract tracing, immunolabelling for mCherry and optical tissue clearing using iDISCO+. Light sheet microscopy was used to image mCherry-positive neurons (561nm) and autofluorescent background (488nm laser). IMARIS software was used to segment and pseudocolor mCherry-positive cells according to brain region.
